# Body Mass Index and Long-Term Follow-Up Outcomes in Patients With Acute Myocardial Infarction by the Median of Non-HDL Cholesterol: Results From an Observational Cohort Study in China

**DOI:** 10.3389/fcvm.2021.750670

**Published:** 2021-10-11

**Authors:** Hui Gao, Aidong Shen, Hui Chen, Hongwei Li

**Affiliations:** ^1^Department of Cardiology, Cardiovascular Center, Beijing Friendship Hospital, Capital Medical University, Beijing, China; ^2^Department of Internal Medical, Medical Health Center, Beijing Friendship Hospital, Capital Medical University, Beijing, China; ^3^Beijing Key Laboratory of Metabolic Disorder Related Cardiovascular Disease, Beijing, China

**Keywords:** obesity, obesity paradox, non-HDL cholesterol, acute myocardial infarction, outcomes

## Abstract

**Background:** The association between obesity, non-HDL cholesterol, and clinical outcomes in subjects with acute myocardial infarction (AMI) undergoing percutaneous coronary intervention (PCI) is incompletely understood. The aim of this investigation was to explore the association between body mass index (BMI), non-high density lipoprotein (non-HDL) cholesterol, and long-term follow-up prognosis.

**Methods:** This present study used data obtained by the Cardiovascular Center of Beijing Friendship Hospital Database Bank. We identified 3,780 consecutive AMI populations aged 25–93 years from 2013 to 2020. Participants were categorized as normal weight (18.5 ≤ BMI <22.9 kg/m^2^), overweight (23.0 ≤ BMI <24.9 kg/m^2^), obese class I (25.0 ≤ BMI <29.9 kg/m^2^), and obese class II (BMI ≥ 30.0 kg/m^2^). The endpoint of interest was cardiovascular (CV) death, all-cause death, myocardial infarction (MI), stroke, unplanned revascularization, and cardiac hospitalization.

**Results:**Participants with higher BMI were younger and more likely to be males compared with lower BMI groups. Elevated non-HDL cholesterol was present in 8.7, 11.0, 24.3, and 5.9% of the normal, overweight, obese class I, and obese class II groups, respectively. After multivariate adjustment, compared to normal-weight participants with decreased non-HDL cholesterol (reference group), obese participants with and without elevated non-HDL cholesterol had a lower risk of mortality (with obese class I and elevated non-HDL cholesterol: hazard ratio [HR] 0.44, 95% confidence interval [CI] 0.28–0.67; with obese class I and decreased non-HDL cholesterol: HR, 0.68, 95% CI, 0.47–0.98; with obese class II and elevated non-HDL cholesterol: HR, 0.42, 95% CI, 0.20–0.87; with obese class II and decreased non-HDL cholesterol: HR, 0.35, 95% CI, 0.16–0.72).

**Conclusion:** In AMI participants performing with PCI, obesity had a better long-term prognosis which probably unaffected by the level of non-HDL cholesterol.

## Introduction

Lipid-lowing therapy plays a pivotal role in reducing mortality of acute myocardial infarction (AMI). Although the level of low-density lipoprotein cholesterol (LDL-C) is reduced, the risk of cardiovascular still remained. Non-high density lipoprotein (non-HDL) cholesterol is relevant to the residual risks of AMI (1). A study enrolled 1,843 subjects demonstrated that lower non-HDL cholesterol levels could estimate lower recurrence rate of AMI in old patients with myocardial infarction (2). Zhu et al. (3) revealed that non-HDL cholesterol was more associated with atherosclerosis when compared with LDL-C in subjects with stable coronary artery disease.

The prevalence of obesity is increasing worldwide. A phenomenon called the “obesity paradox” was investigated in recent years. Increasing evidence showed that lower cardiovascular risk was observed in overweight and obese patients when compared to normal body mass index (BMI) subjects (4). In a Korean national cohort study, obese patients with AMI had a better prognosis than those with normal weight. A survival benefit was also reported in patients with high BMI in several studies (5, 6). However, the explanation of this phenomenon was not well-established.

Most previous studies have emphasized either BMI or non-HDL cholesterol in AMI patients. The relevant researches about the relationship between obesity and cardiovascular prognosis according to non-HDL cholesterol status are limited. Therefore, we sought to investigate the long-term outcomes in overweight and obese subjects with AMI by the median of non-HDL cholesterol.

## Patients and Methods

### Study Design and Participants Enrolment

This study used data from the Cardiovascular Center of Beijing Friendship Hospital Database (CBD) Bank. The CBD Bank is a dataset with a sample of 5,170 consecutive AMI population from January 2013 to August 2020. We excluded patients who were underweight (BMI <18.5 kg/m^2^) due to its small sample size at admission. The patient's clinical and follow-up records were not available for analysis were excluded. Patients with acute infectious diseases, a history of cancer, immune disorders were also excluded. Of the 5,170 patients, 3,780 were recruited in our study. All subjects were followed up to the end of the study duration (August 31, 2020). The median follow-up period is 36.7 months. This retrospective project was approved by the institutional review board of Beijing Friendship Hospital affiliated to Capital Medical University. All methods were carried out in accordance with the ethical standards of the institutional and the Declaration of Helsinki.

### Outcomes and Covariates

BMI was measured based on height, weight and defined as body weight (kg) divided by the squared value of height (m). BMI was classified into four groups on the basis of Asian-specific criteria (7): normal weight (18.5 ≤ BMI <22.9 kg/m^2^), overweight (23.0 ≤ BMI <24.9 kg/m^2^), obese class I (25.0 ≤ BMI < 29.9 kg/m^2^), and obese class II (BMI ≥ 30.0 kg/m^2^). Fasting plasma levels of LDL-C, total cholesterol (TC), high-density lipoprotein (HDL) cholesterol, and triglycerides were directly measured by standard hospital assays at admission. Non-HDL cholesterol was calculated as TC minus HDL cholesterol. Elevated non-HDL cholesterol was defined as ≥3.42 mmol/L according to the median calculation. Briefly, patients were divided into eight groups according to both BMI and non-HDL cholesterol categories. The definition of AMI is in accordance with the Fourth Universal Definition of Myocardial Infarction (8). Only those who adhered to type 1 AMI were recruited in our analysis.

A detailed description of demographic characteristics, biochemical tests, and clinical data was provided from CBD Bank. Smoking and drinking status were estimated from participants' self-reported. Diabetes was defined as medical history, treated with antidiabetic medicines, fasting blood glucose level ≥ 126 mg/dL (7.0 mmol/L), or non-fasting blood glucose level ≥ 200 mg/dL (11.1 mmol/L). Hypertension was defined as the previous history, prescribed with antihypertensive drugs, or systolic blood pressure (SBP) ≥ 140 mmHg or diastolic blood pressure (DBP) ≥ 90 mmHg which were measured at least three times, not on the same day. Echocardiographic, medications at discharge, and coronary angiography results were collected from all participants.

All individuals were followed up with telephone inquiries by trained clinicians. Outcomes included cardiovascular (CV) death, all-cause death, non-fatal myocardial infarction (MI), non-fatal stroke, unplanned revascularization, and cardiac hospitalization were recorded in CBD Bank. CV death was defined as death due to cardiovascular reasons. All-cause death was defined as either cardiac or non-cardiac causes for death. Non-fatal stroke was defined as brain dysfunction examined by brain imaging. The outcome of unplanned revascularization was defined as revascularization of narrowed coronary arteries out of the plan. Cardiac hospitalization was defined as being hospitalized due to symptoms, signs, or objective evidence of heart failure.

### Statistical Analysis

Continuous variables were presented as mean ± SD or median (interquartile range), which were compared using one-way ANOVA (analysis of variance). Categorical variables were reported as counts (percentages), which were compared using chi-square or Fisher's exact test. Cox proportional hazards model was performed to determine the relation of BMI with the prognosis according to the median of non-HDL cholesterol. Normal weight and the concentration of non-HDL cholesterol < 3.42 mmol/L as the reference values for the model. Hazard ratio (HR) and 95% confidence interval (CI) were estimated adjusting for potential confounders included sex, age, smoke, multi-vessel disease, DBP, echocardiography tests [left atrial diameter (LA), and left ventricular end-diastolic diameter (LVEDD)], discharge medications [angiotensin-converting enzyme inhibitor (ACEI), angiotensin receptor blocker (ARB), calcium channel blocker (CCB), and statin] for analyses. Analyses were also stratified according to age group (< 65 and ≥65 years), patterns of AMI [ST-segment elevation myocardial infarction (STEMI) and non-ST-segment elevation myocardial infarction (NSTEMI)], sex, and smoker. BMI as a continuous variable was fitted to a restricted cubic spline with three knots, which described the non-linear relationship with all outcomes in our study. Distributions of BMI category by non-HDL cholesterol were presented with histograms and density plots. We also analyzed the incidence rates as the ratio of the number of adverse events. All analyses were conducted using R Programming Language and SPSS version 25.0 (IBM Inc, Armonk, New York). All reported *p*-values are two-tailed, and *p* < 0.05 was considered to be statistically significant.

## Results

A total of 3,780 individuals with baseline non-HDL cholesterol and BMI calculations were enrolled in this present analysis. Baseline, procedural, and clinical characteristics according to BMI categories by non-HDL cholesterol level were summarized in Table 1. The majority of individuals were obese (56.1%), while only 9.3% had normal weight. The mean age was 63.2 years, and 23.8% were women. Individuals with higher BMI were younger and more probably to be males, had higher TG, and were more frequently prescribed with CCB, ACEI, or ARB at discharge than lower BMI groups. Among patients with a decreased level of non-HDL cholesterol (< 3.42 mmol/L) increasing BMI was less probably to be smokers, drinkers, diagnosed with STEM, and had a history of peripheral vascular disease, which was not observed in those with an elevated level of non-HDL cholesterol (≥3.42 mmol/L).

**Table 1:** Baseline characteristics of study groups.

	**Non-HDL cholesterol** **<** **3.42 mmol/L (*****n*** **=** **1,886)**					**Non-HDL cholesterol** **≥** **3.42 mmol/L (*****n*** **=** **1,894)**				
	**Normal weight**	**Overweight**	**Obese class I**	**Obese class II**	***p*-value**	**Normal weight**	**Overweight**	**Obese class I**	**Obese class II**	***p*-value**
	**(*n* = 432)**	**(*n* = 478)**	**(*n* = 788)**	**(*n* = 188)**		**(*n* = 331)**	**(*n* = 419)**	**(*n* = 919)**	**(*n* = 225)**	
**Demographics**										
Age	68.1 ± 11.1	66.0 ± 11.1	64.4 ± 11.0	61.8 ± 12.9	<0.001	64.7 ± 11.6	62.8 ± 11.0	60.0 ± 11.5	56.0 ± 12.9	<0.001
Male	308 (71.3)	390 (81.6)	632 (80.2)	142 (75.5)	<0.001	212 (64.0)	323 (77.1)	701 (76.3)	172 (76.4)	<0.001
STEMI	233 (53.9)	216 (45.2)	405 (51.4)	78 (41.5)	0.005	178 (53.8)	223 (53.2)	468 (50.9)	106 (47.1)	0.385
SBP, mmHg	125.8 ± 21.6	128.1 ± 21.3	128.4 ± 22.0	134.2 ± 23.0	<0.001	128.5 ± 21.3	130.0 ± 22.3	129.2 ± 21.5	132.5 ± 19.8	0.155
DBP, mmHg	71.2 ± 12.4	72.9 ± 12.0	73.6 ± 12.8	75.8 ± 13.9	<0.001	72.8 ± 11.3	75.1 ± 12.6	74.9 ± 12.6	78.2 ± 13.2	<0.001
Heart rate, bpm	73.4 ± 17.2	73.9 ± 14.8	73.4 ± 14.5	73.4 ± 12.5	0.931	74.9 ± 13.7	75.3 ± 14.8	74.2 ± 13.3	76.4 ± 14.3	0.157
**Past medical history**										
Current smokers	193 (44.7)	209 (43.7)	339 (43.0)	77 (41.0)	0.002	150 (45.3)	223 (53.2)	491 (53.4)	131 (58.2)	0.021
Alcohol use	148 (34.3)	203 (42.5)	301 (38.2)	60 (31.9)	0.015	112 (33.8)	160 (38.2)	377 (41.0)	96 (42.7)	0.081
Hypertension	263 (60.9)	299 (62.6)	556 (70.6)	150 (79.8)	<0.001	187 (56.5)	235 (56.1)	589 (64.1)	165 (73.3)	<0.001
Diabetes	137 (31.7)	174 (36.4)	253 (32.1)	79 (42.0)	0.031	110 (33.2)	123 (29.4)	304 (33.1)	60 (26.7)	0.183
CKD	36 (8.3)	14 (2.9)	40 (5.1)	11 (5.9)	0.004	14 (4.2)	10 (2.4)	28 (3.0)	8 (3.6)	0.531
PAD	38 (8.8)	38 (7.9)	46 (5.8)	5 (2.7)	0.018	13 (3.9)	17 (4.1)	33 (3.6)	7 (3.1)	0.930
Stroke	78 (18.1)	75 (15.7)	129 (16.4)	40 (21.3)	0.313	51 (15.4)	46 (11.0)	109 (11.9)	27 (12.0)	0.281
**Laboratory values**										
TC, mmol/L	3.7 ± 0.5	3.6 ± 0.5	3.6 ± 0.5	3.6 ± 0.5	0.646	5.1 ± 0.6	5.2 ± 0.8	5.2 ± 0.8	5.2 ± 0.9	0.671
TG, mmol/L	1.0 (0.7,1.3)	1.1 (0.8,1.6)	1.2 (1.0,1.7)	1.4 (1.0,2.0)	<0.001	1.6 (1.2,2.0)	1.7 (1.3,2.2)	1.8 (1.4,2.5)	1.9 (1.4,2.7)	<0.001
LDL-C,mmol/L	2.0 ± 0.4	2.0 ± 0.4	2.0 ± 0.4	2.0 ± 0.4	0.220	3.1 ± 0.5	3.1 ± 0.5	3.1 ± 0.6	3.1 ± 0.6	0.762
HDL-C,mmol/L	1.1 ± 0.3	1.0 ± 0.2	1.0 ± 0.2	1.0 ± 0.2	<0.001	1.1 ± 0.2	1.1 ± 0.2	1.0 ± 0.2	1.0 ± 0.2	<0.001
Non-HDL-C,mmol/L	2.6 ± 0.5	2.7 ± 0.5	2.7 ± 0.5	2.7 ± 0.5	0.055	4.1 ± 0.6	4.2 ± 0.7	4.2 ± 0.8	4.3 ± 0.9	0.022
Glycated hemoglobin, %	6.3 ± 1.4	6.4 ± 1.3	6.4 ± 1.3	6.7 ± 1.5	0.006	6.6 ± 1.5	6.7 ± 1.8	6.6 ± 1.5	6.8 ± 1.5	0.653
Creatinine, μmol/L	79.6 (70.1,94.3)	79.7 (70.5,92.6)	81.9 (70.4,94.6)	81.5 (70.5,94.0)	0.928	77.6 (67.0,91.0)	81.0 (69.2,91.3)	80.1 (70.0,91.0)	81.5 (71.5,93.8)	0.302
Peak of CK-MB, ng/ml	56.9 (8.3,109.0)	78.5 (9.1,109.0)	77.5 (11.0,121.0)	68.4 (12.5,109.7)	0.349	86.0 (14.5,161.0)	85.3 (10.0,145.0)	89.1 (15.4,146.0)	72.7 (11.7,123.0)	0.615
Peak of TnI, ng/ml	6.0 (0.7,12.0)	8.9 (0.9,12.0)	8.2 (1.1,12.0)	5.7 (1.6,12.0)	0.417	7.9 (1.6,14.3)	8.2 (1.1,14.4)	9.0 (1.7,14.6)	7.2 (1.5,13.3)	0.817
**Echocardiographic values**										
LA, cm	3.6 ± 0.4	3.7 ± 0.4	3.8 ± 0.4	4.0 ± 0.5	<0.001	3.5 ± 0.4	3.6 ± 0.4	3.7 ± 0.4	3.9 ± 0.4	<0.001
LVEDD, cm	5.1 ± 0.5	5.2 ± 0.5	5.2 ± 0.5	5.4 ± 0.5	<0.001	5.0 ± 0.5	5.1 ± 0.5	5.2 ± 0.5	5.3 ± 0.5	<0.001
LVEF, %	58.1 ± 10.3	59.5 ± 9.7	59.1 ± 9.1	58.9 ± 9.0	0.157	58.7 ± 10.3	58.7 ± 9.3	59.7 ± 8.8	58.9 ± 9.1	0.131
**Angiography values**										
Multi-vessel	296 (68.5)	350 (73.2)	607 (77.0)	140 (74.5)	0.014	224 (67.7)	309 (73.7)	683 (74.3)	179 (79.6)	0.016
LM	36 (8.3)	37 (7.7)	60 (7.6)	12 (6.4)	0.870	30 (9.1)	28 (6.7)	53 (5.8)	15 (6.7)	0.235
LAD	415 (96.1)	456 (95.4)	758 (96.2)	180 (95.7)	0.914	312 (94.3)	406 (96.9)	892 (97.1)	214 (95.1)	0.083
Pre-PCI TIMI 0/1 flow	143 (33.1)	161 (33.7)	286 (36.3)	65 (34.6)	0.662	115 (34.7)	176 (42.0)	401 (43.6)	90 (40.0)	0.042
Post-PCI TIMI 3 flow	327 (75.7)	356 (74.5)	605 (76.8)	135 (71.8)	0.502	251 (75.8)	329 (78.5)	705 (76.7)	170 (75.6)	0.784
**Medications at discharge**										
Aspirin	400 (92.6)	456 (95.4)	754 (95.7)	181 (96.3)	0.079	316 (95.5)	405 (96.7)	885 (96.3)	218 (96.9)	0.798
Clopidogrel	311 (72.0)	341 (71.3)	571 (72.5)	136 (72.3)	0.978	245 (74.0)	319 (76.1)	674 (73.3)	174 (77.3)	0.526
ACEI or ARB	258 (59.7)	330 (69.0)	540 (68.5)	154 (81.9)	<0.001	198 (59.8)	272 (64.9)	643 (70.0)	172 (76.4)	<0.001
Beta-blockers	296 (68.5)	337 (70.5)	592 (75.1)	148 (78.7)	0.013	266 (80.4)	315 (75.2)	715 (77.8)	187 (83.1)	0.092
CCB	64 (14.8)	78 (16.3)	179 (22.7)	62 (33.0)	<0.001	57 (17.2)	70 (16.7)	162 (17.6)	64 (28.4)	0.001
Statins	384 (88.9)	427 (89.3)	710 (90.1)	169 (89.9)	0.918	300 (90.6)	381 (90.9)	825 (89.8)	197 (87.6)	0.558
Length of stay, days	8.0 (6.0,10.0)	8.0 (6.0,10.0)	7.0 (6.0,10.0)	7.0 (6.0,9.0)	0.116	8.0 (6.0,10.0)	8.0 (6.0,10.0)	8.0 (6.0,10.0)	8.0 (6.0,9.0)	0.680

*Normal weight: 18.5–22.9 kg/m^2^; Overweight: 23.0–24.9 kg/m^2^; Obese class I: 25.0–29.9 kg/m^2^; Obese class II: ≥30.0 kg/m^2^*.

*Values are presented as mean ± SD, median (IQR) or number (%)*.

*STEMI, ST-elevation myocardial infarction; BMI, body mass index; SBP, systolic blood pressure; DBP, diastolic blood pressure; CKD, chronic kidney disease; PAD, peripheral artery disease; TG, triglyceride; HDL-C, high-density lipoprotein cholesterol; PCI, percutaneous coronary intervention; ACEI or ARB, angiotensin-converting enzyme inhibitor/angiotensin receptor blocker; CCB, calcium channel blocker; eGFR, estimated glomerular filtration rate; TC, total cholesterol; LDL-C, low-density lipoprotein cholesterol; MI, myocardial infarction; LA, left atrial diameter; LVEDD, left ventricular end diastolic dimension; LAD, left anterior descending branch; LVEF, left ventricular ejection fraction; LM, left main coronary artery*.

**Figure 1:**
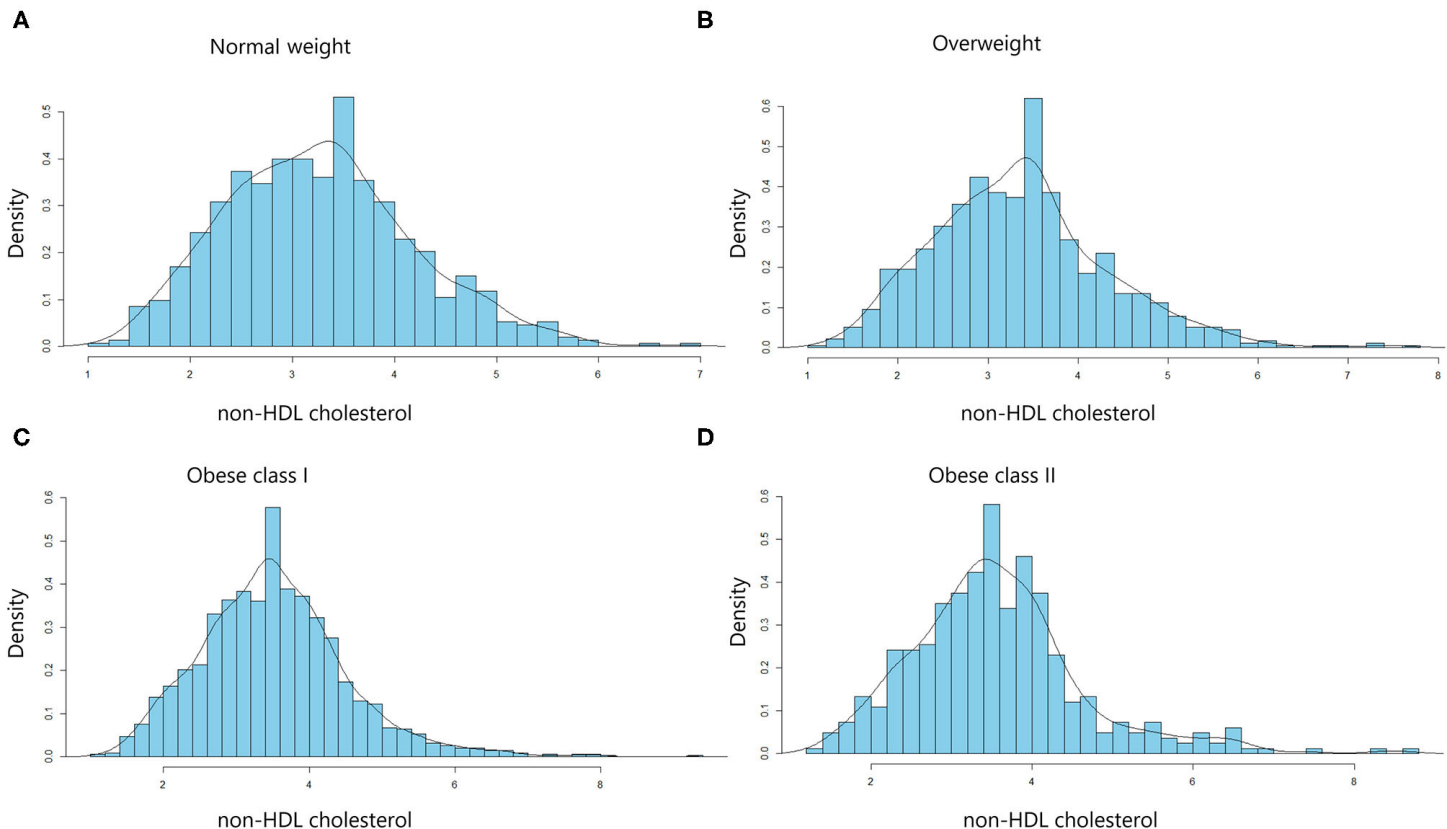
Distribution of non-HDL cholesterol levels in the different body mass index (BMI) categories. Histograms for non-HDL cholesterol distribution in normal-weight **(A)**, overweight **(B)**, obese class I **(C)**, and obese class II **(D)** subjects. Values are density (%).

The distribution of non-HDL cholesterol by BMI was described in Figure 1. Median non-HDL cholesterol concentrations were 3.24 mmol/L (interquartile range, 2.58–3.84 mmol/L) for normal weight participants, 3.34 mmol/L (interquartile range, 2.68–3.89 mmol/L) for overweight participants, 3.43 mmol/L (interquartile range, 2.83–4.04 mmol/L) for obese I class participants, and 3.46 mmol/L (interquartile range, 2.85–4.05 mmol/L) for obese II class participants.

**Figure 2:**
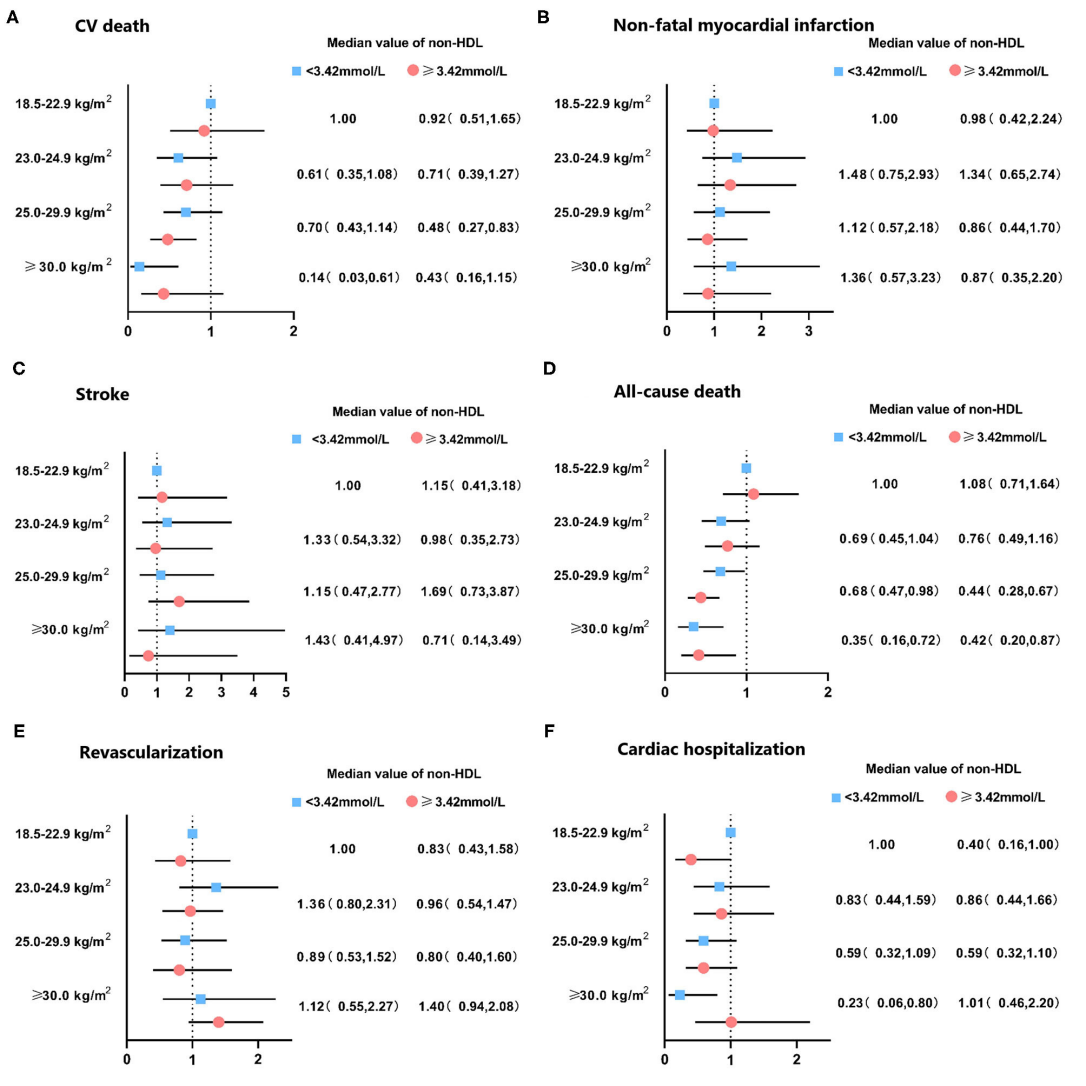
Hazard ratio of clinical outcomes in patients with acute coronary syndrome by non-HDL cholesterol levels and body mass index. Adjusted for sex, age, smoke, multi-vessel disease, DBP, LA, LVEDD, ACEI/ARB, CCB, statin. DBP, diastolic blood pressure; LA, left atrial diameter; LVEDD, left ventricular end diastolic dimension; ACEI/ARB, angiotensin-converting enzyme inhibitor/angiotensin receptor blocker; CCB, calcium channel blocker; CV, cardiovascular.

**Figure 3:**
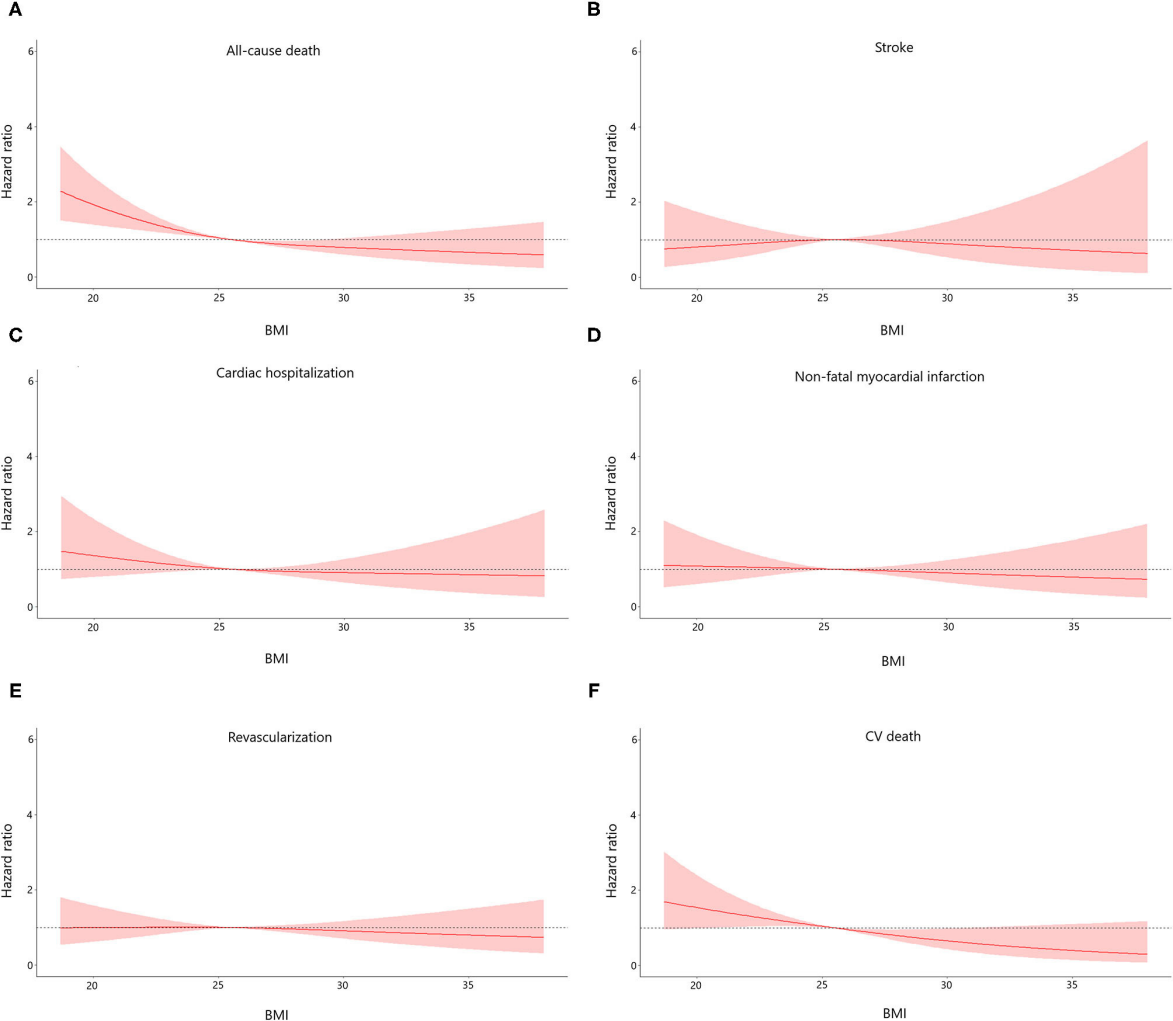
Association between body mass index (BMI) and the risk of all endpoints of the study population. Adjusted for sex, age, smoke, multi-vessel disease, DBP, LA, LVEDD, ACEI/ARB, CCB, statin. DBP, diastolic blood pressure; LA, left atrial diameter; LVEDD, left ventricular end diastolic dimension; ACEI/ARB, angiotensin-converting enzyme inhibitor/angiotensin receptor blocker; CCB, calcium channel blocker.

The median follow-up period was 36.7 months (interquartile range: 36.3–37.2 months). A total number of 298 (7.9) subjects suffered from the death of which 162 (4.3) were CV deaths. Individuals with normal weight and decreased level of non-HDL cholesterol had the highest incidence of all-cause and CV death among all groups. Stepwise higher risk of revascularization was related with stepwise higher BMI categories in an elevated level of non-HDL cholesterol. However, there was a stepwise decrease in the risk of cardiac rehospitalization from the normal to the overweight, obese class I, and obese class II group (4.6, 4.0, 3.2, and 1.6%, respectively; Table 2). The risks of clinical results included non-fatal stroke, CV and all-cause death, cardiac-rehospitalization, non-fatal MI, and revascularization by BMI and non-HDL cholesterol in AMI participants are represent in Supplementary Table 1. When compared with participants with non-HDL cholesterol < 3.42 mmol/L and normal weight (reference group), individuals with non-HDL cholesterol ≥3.42 mmol/L had hazard ratios for all-cause death of 0.32 (95% CI, 0.21–0.49) for obese class I, and 0.29 (95% CI, 0.14–0.59) for obese class II participants. Similar results were also observed in the risk of CV death. In subjects with a decreased level of non-HDL cholesterol, the obese class II was associated with a lower risk of CV (HR, 0.13, 95% CI, 0.03–0.57) and all-cause death (HR, 0.36, 95% CI, 0.17–0.73), respectively, when compared to normal weight. The relationship between all endpoints across a range of BMI after adjustment of sex, age, smoking status, multi-vessel disease, DBP, echocardiography (LA, EDD), medications at discharge (ACEI/ARB, CCB, statin) was revealed in Figure 2. In multivariable-adjusted regression analysis, obese class I (HR, 0.68, 95% CI, 0.47–0.98 for decreased non-HDL cholesterol, HR, 0.44, 95% CI, 0.28–0.67 for increased non-HDL cholesterol) and obese class II (HR, 0.35, 95% CI, 0.16–0.72 for decreased non-HDL cholesterol, HR, 0.42, 95% CI, 0.20–0.87 for increased non-HDL cholesterol) subjects remained significant lower risk of death compared to the reference group, regardless of non-HDL cholesterol. However, after adjustment for confounders, obese class II participants with elevated non-HDL cholesterol revealed a similar risk of CV death to the reference group. Besides, there were no associations between non-HDL cholesterol and risk of non-fatal MI, cardiac rehospitalization, revascularization, non-fatal stroke at any category of BMI. Figure 3 shows associations between BMI and clinical endpoints with near-linear decreases in adjusted HR with higher BMI.

**Table 2:** Comparison of clinical outcomes among BMI categories.

	**All**	**Non-HDL cholesterol** **<** **3.42 mmol/L (*****n*** **=** **1,886)**					**Non-HDL cholesterol** **≥** **3.42 mmol/L (*****n*** **=** **1,894)**				
		**Normal weight**	**Overweight**	**Obese class I**	**Obese class II**	***p*-value**	**Normal weight**	**Overweight**	**Obese class I**	**Obese class II**	***p*-value**
		**(*n* = 432)**	**(*n* = 478)**	**(*n* = 788)**	**(*n* = 188)**		**(*n* = 331)**	**(*n* = 419)**	**(*n* = 919)**	**(*n* = 225)**	
CV death	162 (4.3)	31 (7.2)	21 (4.4)	41 (5.2)	2 (1.1)	0.013	19 (5.7)	19 (4.5)	24 (2.6)	5 (2.2)	0.025
All-cause death	298 (7.9)	53 (12.3)	42 (8.8)	69 (8.8)	9 (4.8)	0.005	40 (12.1)	37 (8.8)	39 (4.2)	9 (4.0)	<0.001
Non-fatal MI	142 (3.8)	13 (3.0)	24 (5.0)	30 (3.8)	10 (5.3)	0.357	10 (3.0)	19 (4.5)	28 (3.0)	8 (3.6)	0.546
Revascularization	233 (6.2)	22 (5.1)	39 (8.2)	43 (5.5)	14 (7.4)	0.154	16 (4.8)	26 (6.2)	58 (6.3)	15 (6.7)	0.769
Non-fatal stroke	78 (2.1)	8 (1.9)	12 (2.5)	15 (1.9)	4 (2.1)	0.880	7 (2.1)	7 (1.7)	23 (2.5)	2 (0.9)	0.433
Cardiac rehospitalization	126 (3.3)	20 (4.6)	19 (4.0)	25 (3.2)	3 (1.6)	0.246	6 (1.8)	17 (4.1)	25 (2.7)	11 (4.9)	0.116

*P-values for differences among BMI groups*.

*Normal weight: 18.5–22.9 kg/m^2^; Overweight: 23.0–24.9 kg/m^2^; Obese class I: 25.0–29.9 kg/m^2^; Obese class II: ≥30.0 kg/m^2^*.

**Figure 4:**
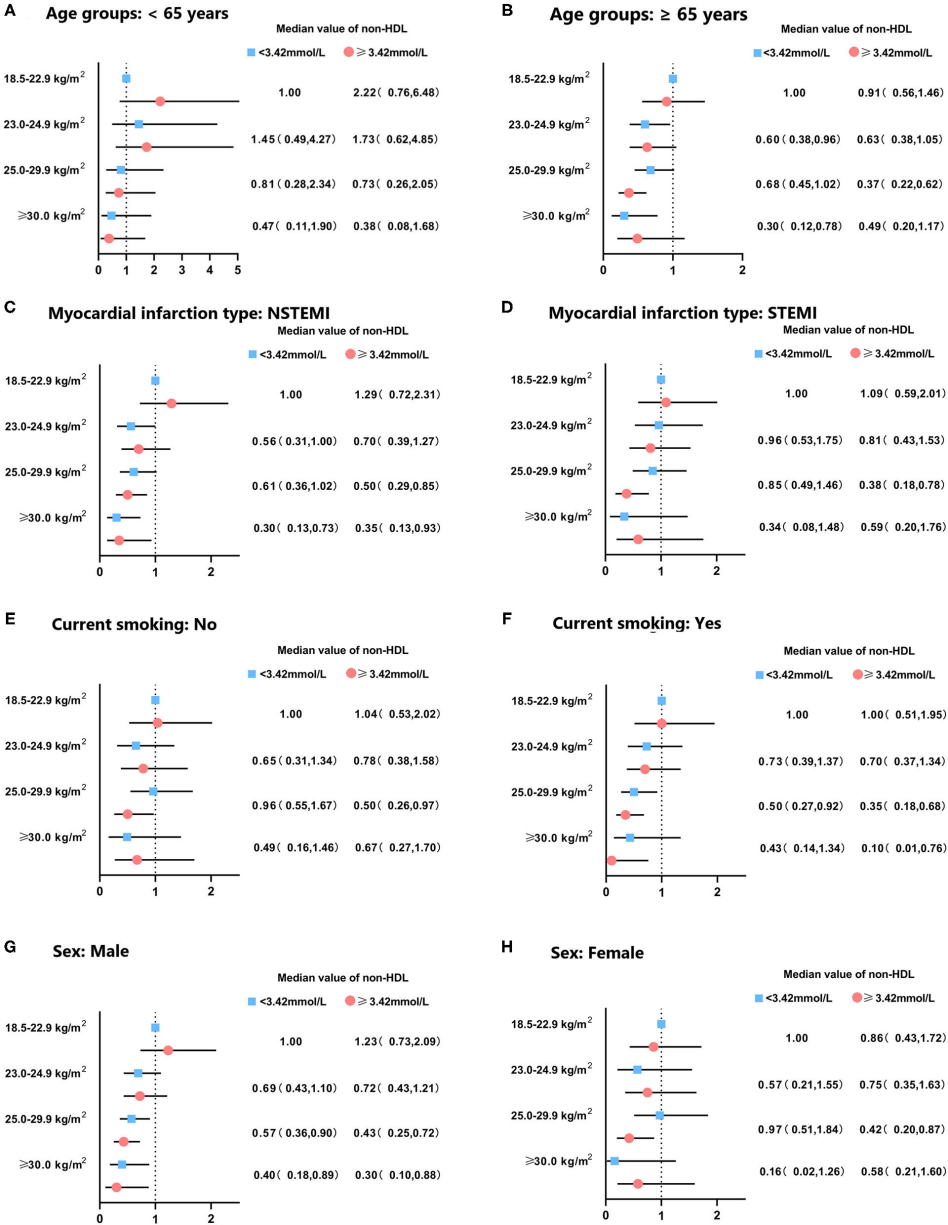
Subgroup analysis for hazard ratio of mortality in patients with acute coronary syndrome according to body mass index and non-HDL cholesterol levels. Subgroup was stratified by **(A,B)** age, **(C,D)** clinical diagnosis of AMI, **(E,F)** smoking status, and **(G,H)** sex. Adjusted for sex, age, smoke, multi-vessel disease, DBP, LA, LVEDD, ACEI/ARB, CCB, statin.

When participants were categorized by sex, age, clinical diagnosis, and smoking status, obese class I participants with elevated non-HDL cholesterol had a lower risk of mortality in both males and females, but only significant in participants ≥65 years. Males with BMI ≥ 25 kg/m^2^ had a significantly lower risk of mortality than normal-weight subjects (all *p* < 0.05) (Figure 4).

## Discussion

About four-fifths of individuals with AMI were overweight or obese in our study. Class II obesity (BMI ≥ 30 kg/m^2^) affects 1 in 10 individuals and was more common in men than women. This analysis revealed that obesity and non-HDL cholesterol affected the long-term clinical outcomes of 3,780 individuals with AMI. The “obesity paradox” was observed in AMI individuals. Obese decreased the risk of all-cause death in participants with a decreased or increased level of non-HDL cholesterol. These were more apparent in males, age ≥ 65 years, current smokers, and present with NSTEMI. CV death seemed to be lowered in BMI ≥30.0 kg/m^2^ patients with non-HDL cholesterol <3.42 mmol/L, while it can only be observed in BMI 25.0–29.9 kg/m^2^ patients with non-HDL cholesterol ≥ 3.42 mmol/L. Obesity decreased the risk of cardiac rehospitalization in subjects with a lower level of non-HDL cholesterol, while increased the rate of revascularization in subjects with a higher level of non-HDL cholesterol.

### Obesity Paradox

Weight gain is a risk factor for cardiovascular disease. In a large-scale, population-based longitudinal analysis of 2,611,450 subjects aged 20–39 years reported that obesity was associated with an elevated risk of coronary artery disease (9). However, in contrast to the previous study, young populations with STEMI and BMI ≥ 40 kg/m^2^ experience less MI, and have a better cardiac function (10). In a study with 2,238 patients who underwent primary percutaneous coronary intervention (pPCI) for STEMI, BMI was not associated with 1-year mortality rate or cardiac hospitalization (11). Obesity is generally observed in subjects with acute coronary symptom, while they usually have a better prognosis after coronary intervention (12). In a meta-analysis study, the obesity paradox has been recognized in subjects with post-percutaneous coronary intervention (13). The higher BMI was associated with a lower risk of mortality in female patients with PCI, except for the severely obese population (14). The HUNT study revealed that the risk of MI among obese patients was not significantly increased by comparison with normal-weight patients (12). In particular, the association of obesity has also been identified in patients younger than 65 years old in this research. Multiple mechanisms have been put forward to explain the association between obesity and atherosclerosis. Subcutaneous fat accumulates in fat tissue, which may reduce the increased catabolism in neurohormonal pathways (15). Several factors are also involved in the pathophysiological process, such as oxidative stress-related factors and inflammatory cytokines (16). The risk of mortality in extremely obese (BMI ≥ 40 kg/m^2^) subjects presenting with STEMI is increased, while the underlying mechanism remains unclear (10). Prothrombotic state is one of the interpretations for this phenomenon, which may contribute to the adverse event (17).

### Obesity Paradox in Non-HDL Cholesterol

Non-HDL cholesterol is the cholesterol content of all apoB particles, i.e., LDL, very-low-density lipoproteins (VLDL), intermediate-density lipoprotein, and lipoprotein (a) (18). A simple way to estimate non-HDL cholesterol is to calculate it as total cholesterol minus HDL cholesterol. During atherosclerotic plaque formation and progression, non-HDL cholesterol plays an essential role. Previous studies have shown non-HDL cholesterol could be used as one of the risk indicators in atherosclerotic cardiovascular disease (ASCVD) assessment, especially in high risk populations (19, 20). In a meta-analysis, non-HDL cholesterol has been proved more predictive than LDL-C in subjects with hypertriglyceridemia (21). Non-HDL-cholesterol is convenient and reliable, while it is not well recognized and widely used. It is also unclear whether non-HDL cholesterol affects the correlation between obesity and cardiovascular endpoints.

Dyslipidemia is recognized as one of the obesity-associated metabolic disorders, which increases the risk of cardiovascular events (22). In our analysis, we found that CV death was lower in BMI, 25.0–29.9 kg/m^2^ patients with non-HDL cholesterol ≥3.42 mmol/L. This group was less likely to be current smokers, diagnosed with a history of hypertension, had multi vessel disease compared with those in BMI ≥30 kg/m^2^. It could be one of the underlying causes of this phenomenon. In terms of CV death, the benefits of obesity were weakened in obese class II patients due to elevated level of non-HDL cholesterol. Not all obese subjects were observed with dyslipidemia, however, the prevalence of hypertriglyceridemia is high in abdominally obese individuals (23). Besides, elevated LDL-C and reduced HDL-C levels could be observed in subjects with obesity (24). This metabolism might be a transient condition that exists in a large obese population (25). Obesity was observed due to the redundant cumulation of intramuscular triglyceride and other metabolic factors (26, 27). In a large-scale population analysis, metabolically healthy women with BMI ≥25 kg/m^2^ had a higher risk than normal-weight women (28). However, the progress of dyslipidemia had little effect on the risk for obese women without metabolically unhealthy at the beginning of the study. Mørkedal et al. (12) revealed that only a moderately increased risk of AMI among metabolically healthy populations with BMI > 40 kg/m^2^. Data from meta-analyses revealed that increasing BMI not decreased the risk of cardiovascular endpoints during follow-up time in subjects without dyslipidemia, hypertension, or diabetes (29, 30). In the study with 61,299 Norse free of cardiovascular disease, metabolically unhealthy status was related to the risk of AMI and without difference among categories of BMI (12). Previous studies showed similar results with a long-term follow-up period of time (31–33).

### Limitations

There are some limitations in this present study. First, the involved individuals in the cohort were limited due to the single-center research. Second, BMI was collected based on self-reported data, in which participants reported their weight and height without precise measurements. Thirdly, we did not have data on weight change, waist circumference, fat mass, or waist-to-hip ratio, which has been declared the prediction of mortality in individuals with cardiovascular disease (34–36). Some potential confounders were not available which may perform to adjust the possibility of residual confounding.

## Conclusion

This study investigates that the increasing numbers of participants who are obese. In individuals present with AMI, obesity had a protective effect on mortality, both in elevated and decreased non-HDL cholesterol levels. Future clinical practices that demonstrate the association of BMI and non-HDL cholesterol with cardiovascular disease are required to illustrate the interpretations of our findings.

## Data Availability Statement

The raw data supporting the conclusions of this article will be made available by the authors, without undue reservation.

## Ethics Statement

The studies involving human participants were reviewed and approved by the Institutional Review Board of Beijing Friendship Hospital affiliated to Capital Medical University. The patients/participants provided their written informed consent to participate in this study.

## Author Contributions

HL designed the protocol of the study. HG draft the manuscript. HG, AS, HC, and HL participated in the collection, interpretation, and analysis of the data. All authors approved the final version for publication.

## Funding

This research was supported by a grant from Beijing Key Clinical Subject Program.

## Conflict of Interest

The authors declare that the research was conducted in the absence of any commercial or financial relationships that could be construed as a potential conflict of interest.

## Publisher's Note

All claims expressed in this article are solely those of the authors and do not necessarily represent those of their affiliated organizations, or those of the publisher, the editors and the reviewers. Any product that may be evaluated in this article, or claim that may be made by its manufacturer, is not guaranteed or endorsed by the publisher.
