# Supplementary material for: Body Mass Index and Long-Term Follow-Up Outcomes in Patients With Acute Myocardial Infarction by the Median of Non-HDL Cholesterol: Results From an Observational Cohort Study in China
Source: Front Cardiovasc Med. 2021 Oct 11;8:750670. doi: 10.3389/fcvm.2021.750670 (PMC8634779; doi:10.3389/fcvm.2021.750670)
Supplement: Supplementary file 1 [file Table_1.DOCX]

**Supplementary Table 1 Risk of Outcomes**

|  | **Events (%)** | **Unadjusted HR (95% CI)** | **p value** | **Adjusted HR (95% CI)** | **p value** |
| --- | --- | --- | --- | --- | --- |
| **CV death** |  |  |  |  |  |
| **Non-HDL cholesterol<3.42 mmol/L** |  |  |  |  |  |
| Normal weight | 31(7.2) | 1.00 | Ref | 1.00 | Ref |
| Overweight | 21(4.4) | 0.57(0.33,1.00) | 0.051 | 0.61(0.35,1.08) | 0.094 |
| Obese class I | 41(5.2) | 0.70(0.44,1.12) | 0.145 | 0.70(0.43,1.14) | 0.162 |
| Obese class II | 2(1.1) | 0.13(0.03,0.57) | 0.006 | 0.14(0.03,0.61) | 0.009 |
| **Non-HDL cholesterol≥3.42 mmol/L** |  |  |  |  |  |
| Normal weight | 19(5.7) | 0.80(0.45,1.42) | 0.451 | 0.92(0.51,1.65) | 0.800 |
| Overweight | 19(4.5) | 0.61(0.34,1.08) | 0.093 | 0.71(0.39,1.27) | 0.251 |
| Obese class I | 24(2.6) | 0.34(0.20,0.58) | <0.001 | 0.48(0.27,0.83) | 0.009 |
| Obese class II | 5(2.2) | 0.28(0.11,0.72) | 0.009 | 0.43(0.16,1.15) | 0.095 |
| **Cardiac rehospitalization** |  |  |  |  |  |
| **Non-HDL cholesterol<3.42 mmol/L** |  |  |  |  |  |
| Normal weight | 20(4.6) | 1.00 | Ref | 1.00 | Ref |
| Overweight | 19(4.0) | 0.82(0.43,1.53) | 0.537 | 0.83(0.44,1.59) | 0.591 |
| Obese class I | 25(3.2) | 0.67(0.37,1.20) | 0.182 | 0.59(0.32,1.09) | 0.095 |
| Obese class II | 3(1.6) | 0.32(0.09,1.09) | 0.069 | 0.23(0.06,0.80) | 0.022 |
| **Non-HDL cholesterol≥3.42 mmol/L** |  |  |  |  |  |
| Normal weight | 6(1.8) | 0.38(0.15,0.95) | 0.039 | 0.40(0.16,1.00) | 0.052 |
| Overweight | 17(4.1) | 0.85(0.44,1.62) | 0.623 | 0.86(0.44,1.66) | 0.662 |
| Obese class I | 25(2.7) | 0.55(0.31,1.00) | 0.052 | 0.59(0.32,1.10) | 0.099 |
| Obese class II | 11(4.9) | 1.00(0.48,2.09) | 0.994 | 1.01(0.46,2.20) | 0.967 |
| **Non‑fatal MI** |  |  |  |  |  |
| **Non-HDL cholesterol<3.42 mmol/L** |  |  |  |  |  |
| Normal weight | 13(3.0) | 1.00 | Ref | 1.00 | Ref |
| Overweight | 24(5.0) | 1.55(0.79,3.06) | 0.198 | 1.48(0.75,2.93) | 0.256 |
| Obese class I | 30(3.8) | 1.23(0.64,2.37) | 0.519 | 1.12(0.57,2.18) | 0.734 |
| Obese class II | 10(5.3) | 1.67(0.73,3.82) | 0.220 | 1.36(0.57,3.23) | 0.474 |
| **Non-HDL cholesterol≥3.42 mmol/L** |  |  |  |  |  |
| Normal weight | 10(3.0) | 1.01(0.44,2.30) | 0.977 | 0.98(0.42,2.24) | 0.963 |
| Overweight | 19(4.5) | 1.46(0.72,2.96) | 0.292 | 1.34(0.65,2.74) | 0.417 |
| Obese class I | 28(3.0) | 0.95(0.49,1.83) | 0.881 | 0.86(0.44，1.70) | 0.676 |
| Obese class II | 8(3.6) | 1.06(0.44,2.57) | 0.886 | 0.87(0.35,2.20) | 0.783 |
| **Revascularization** |  |  |  |  |  |
| **Non-HDL cholesterol<3.42 mmol/L** |  |  |  |  |  |
| Normal weight | 22(5.1) | 1.00 | Ref | 1.00 | Ref |
| Overweight | 39(8.2) | 1.51(0.89,2.55) | 0.120 | 1.36(0.80,2.31) | 0.252 |
| Obese class I | 43(5.5) | 1.05(0.62,1.75) | 0.851 | 0.89(0.53,1.52) | 0.689 |
| Obese class II | 14(7.4) | 1.38(0.70，2.70) | 0.344 | 1.12(0.55,2.27) | 0.737 |
| **Non-HDL cholesterol≥3.42 mmol/L** |  |  |  |  |  |
| Normal weight | 16(4.8) | 0.93(0.49,1.78) | 0.848 | 0.83(0.43，1.58) | 0.572 |
| Overweight | 26(6.2) | 1.17(0.66,2.06) | 0.582 | 0.96(0.54,1.47) | 0.895 |
| Obese class I | 58(6.3) | 1.16(0.71,1.90) | 0.544 | 0.80(0.40,1.60) | 0.650 |
| Obese class II | 15(6.7) | 1.16(0.60,2.24) | 0.652 | 1.40(0.94,2.08) | 0.529 |
| **Non‑fatal stroke** |  |  |  |  |  |
| **Non-HDL cholesterol<3.42 mmol/L** |  |  |  |  |  |
| Normal weight | 8(1.9) | 1.00 | Ref | 1.00 | Ref |
| Overweight | 12(2.5) | 1.27(0.52,3.12) | 0.591 | 1.33(0.54,3.32) | 0.528 |
| Obese class I | 15(1.9) | 0.98(0.41,2.33) | 0.979 | 1.15(0.47,2.77) | 0.753 |
| Obese class II | 4(2.1) | 1.08(0.32,3.60) | 0.894 | 1.43(0.41,4.97) | 0.569 |
| **Non-HDL cholesterol≥3.42 mmol/L** |  |  |  |  |  |
| Normal weight | 7(2.1) | 1.12(0.40,3.09) | 0.823 | 1.15(0.41,3.18) | 0.787 |
| Overweight | 7(1.7) | 0.86(0.31,2.39) | 0.783 | 0.98(0.35,2.73) | 0.971 |
| Obese class I | 23(2.5) | 1.24(0.55,2.78) | 0.592 | 1.69(0.73,3.87) | 0.214 |
| Obese class II | 2(0.9) | 0.41(0.08,1.97) | 0.271 | 0.71(0.14,3.49) | 0.677 |
| **All cause death** |  |  |  |  |  |
| **Non-HDL cholesterol<3.42 mmol/L** |  |  |  |  |  |
| Normal weight | 53(12.3) | 1.00 | Ref | 1.00 | Ref |
| Overweight | 42(8.8) | 0.66(0.44,1.00) | 0.052 | 0.69(0.45,1.04) | 0.079 |
| Obese class I | 69(8.8) | 0.69(0.48,0.99) | 0.046 | 0.68(0.47,0.98) | 0.043 |
| Obese class II | 9(4.8) | 0.36(0.17,0.73) | 0.005 | 0.35(0.16,0.72) | 0.005 |
| **Non-HDL cholesterol≥3.42 mmol/L** |  |  |  |  |  |
| Normal weight | 40(12.1) | 0.98(0.65,1.48) | 0.931 | 1.08(0.71,1.64) | 0.705 |
| Overweight | 37(8.8) | 0.69(0.45,1.05) | 0.086 | 0.76(0.49,1.16) | 0.213 |
| Obese class I | 39(4.2) | 0.32(0.21,0.49) | <0.001 | 0.44(0.28,0.67) | <0.001 |
| Obese class II | 9(4.0) | 0.29(0.14,0.59) | 0.001 | 0.42(0.20,0.87) | 0.019 |

Normal weight: 18.5–22.9 kg/m^2^; Overweight: 23.0–24.9 kg/m^2^; Obese class I: 25.0–29.9 kg/m^2^; Obese class II: ≥30.0 kg/m^2^

Adjusted factors included sex, age, smoke, multi-vessel disease, DBP, LA, EDD, ACEI/ARB, CCB, statin
